# Genome wide evolutionary analyses reveal serotype specific patterns of positive selection in selected *Salmonella *serotypes

**DOI:** 10.1186/1471-2148-9-264

**Published:** 2009-11-14

**Authors:** Yeşim Soyer, Renato H Orsi, Lorraine D Rodriguez-Rivera, Qi Sun, Martin Wiedmann

**Affiliations:** 1 Department of Food Science, Cornell University, 412 Stocking Hall, Ithaca, NY 14853, USA; 2 Computational Biology Service Unit, Life Sciences Core Laboratories Center, Cornell University, 618 Rhodes Hall, Ithaca, NY 14853, USA

## Abstract

**Background:**

The bacterium *Salmonella enterica *includes a diversity of serotypes that cause disease in humans and different animal species. Some *Salmonella *serotypes show a broad host range, some are host restricted and exclusively associated with one particular host, and some are associated with one particular host species, but able to cause disease in other host species and are thus considered "host adapted". Five *Salmonella *genome sequences, representing a broad host range serotype (Typhimurium), two host restricted serotypes (Typhi [two genomes] and Paratyphi) and one host adapted serotype (Choleraesuis) were used to identify core genome genes that show evidence for recombination and positive selection.

**Results:**

Overall, 3323 orthologous genes were identified in all 5 *Salmonella *genomes analyzed. Use of four different methods to assess homologous recombination identified 270 genes that showed evidence for recombination with at least one of these methods (false discovery rate [FDR] <10%). After exclusion of genes with evidence for recombination, site and branch specific models identified 41 genes as showing evidence for positive selection (FDR <20%), including a number of genes with confirmed or likely roles in virulence and *ompC*, a gene encoding an outer membrane protein, which has also been found to be under positive selection in other bacteria. A total of 8, 16, 7, and 5 genes showed evidence for positive selection in Choleraesuis, Typhi, Typhimurium, and Paratyphi branch analyses, respectively. Sequencing and evolutionary analyses of four genes in an additional 42 isolates representing 23 serotypes confirmed branch specific positive selection and recombination patterns.

**Conclusion:**

Our data show that, among the four serotypes analyzed, (i) less than 10% of *Salmonella *genes in the core genome show evidence for homologous recombination, (ii) a number of *Salmonella *genes are under positive selection, including genes that appear to contribute to virulence, and (iii) branch specific positive selection contributes to the evolution of host restricted *Salmonella *serotypes.

## Background

*Salmonella *is a ubiquitous human and animal pathogen. This genus contains >2,500 recognized serotypes and is divided into two species, *Salmonella bongori *and *Salmonella enterica*. *S. enterica *consists of six subspecies (i.e., *enterica, salamae*, *arizonae, diarizonae, houtenae*, and *indica*) [1]. *Salmonella enterica *subsp. *enterica *serotypes can also be divided into subdivisions according to their host adaptation [2]. For example, Uzzau et al. [2] proposed that *Salmonella *serotypes can be divided into (i) host-restricted *Salmonella *serotypes (i.e., serotypes exclusively associated with one particular host, e.g., *Salmonella *Typhi and Paratyphi A); (ii) host-adapted *Salmonella *serotypes (i.e., serotypes prevalent in one particular host species, but able to cause disease in other host species, e.g., *Salmonella *Choleraesuis); and (iii) unrestricted *Salmonella *serotypes (i.e., serotypes capable of causing self-limiting gastroenteritis and, less commonly, systemic disease in a wide range of host species, e.g., *Salmonella *Typhimurium).

Multi-locus sequence typing (MLST) data indicate that the last common ancestor of the human host-adapted *Salmonella *Typhi existed 15,000-150,000 years ago [3]. The evolution of *Salmonella *Typhi towards a lifestyle characterized by systemic infection and transmission by excretion through the gall bladder rather than luminal gut colonization [4] involved a combination of acquisition events (e.g., acquisition of Vi capsule related genes), and deletion events (e.g., loss of virulence-associated genes, such as several genes in SPI-1, SPI-2, SPI-3, SPI-4 and SPI-5). *Salmonella *Paratyphi A also causes typhoid fever, although the symptoms are typically milder than those caused by *Salmonella *Typhi. While *Salmonella *Paratyphi A also appears to have evolved recently, *Salmonella *Typhi and Paratyphi A clearly show distinct differences in their genome evolution, including a number of unique gene inactivation events in these two serotypes [5]. Non-typhoidal *Salmonella *serotypes are responsible for gastroenteritis in humans and other animals. These serotypes are mainly transmitted by ingestion of food, feed, or water contaminated with infected feces [6], but can also be transmitted by direct contact [7,8]. Disease caused by non-typhoidal *Salmonella *is one of the most common bacterial foodborne diseases worldwide [9]. *Salmonella *Typhimurium is one of the most common non-typhoidal *Salmonella *serotypes, is found worldwide, and can cause disease, predominantly self limiting gastroenteritis, in a large number of animal species [2]. The host adapted *Salmonella *Choleraesuis can cause severe disease, characterized by septicemia and enterocolitis, in swine. While relatively uncommon, this serotype can also infect humans where it typically causes severe invasive infections, e.g., infective aneurysm [10].

The importance of acquisition of novel (non-homologous) genes by lateral gene transfer has been clearly demonstrated in a number of bacteria, including a number of bacterial pathogens [11-14]. For example, acquisition of pathogenicity islands has played a critical role in the evolution of *Salmonella *[13] and other Gram-negative and Gram-positive pathogens [15]. Gene degradation and gene deletions also have been shown to play a critical role in bacterial evolution, particularly when organisms with a broad niche specificity adapt to narrow and specific ecological niches [5,16]. For example, it has been suggested that gene degradation and gene deletion contribute to host adaptation in both *Salmonella *Typhi and *Salmonella *Paratyphi A [5]. Microarray technologies have also allowed for rapid and large scale studies on gene presence/absence in a large number of isolates, including in *Salmonella *[17]. In addition to gene acquisition and deletion, positive selection and homologous recombination, play important roles in the evolution of bacteria and bacterial pathogens [18-21].

Genome wide studies on positive selection and homologous recombination in bacterial pathogens, including *Streptococcus *spp. [20], *Listeria monocytogenes *[18], *Campylobacter *[22], *E. coli *[23,24], and *Shigella *[24] have contributed to a better understanding of the evolution of these important pathogens. So far, no genome wide analyses of positive selection in *Salmonella *have been reported. One study [25] evaluated 410 genes present in both *S. enterica *and *E. coli *and reported that 50% of amino acid substitutions in these genes appear to have been fixed by positive selection in one of these species. In order to further improve our understanding of the evolution of *Salmonella*, we performed full genome analyses for homologous recombination and positive selection using the completed and published genome sequences for five *Salmonella *strains, including the host restricted *Salmonella *Typhi (two strains) and Paratyphi A, the host adapted *Salmonella *Choleraesuis, and the broad host range *Salmonella *Typhimurium. Our analyses focused on the evolution of core genome genes (i.e., genes found in all 5 genomes) and did not include efforts to detect genes acquired by *Salmonella *through horizontal gene transfer and subsequent non-homologous recombination (e.g., virulence gene islands), as these types of evolutionary events have already been well characterized [13,26,27]. Analysis of the *Salmonella *serotypes included in our study here will, in particular, provide an improved understanding in the roles of positive selection and homologous recombination in the evolution of host-adapted pathogen strains and lineages.

## Methods

### Genome sequences

Five available annotated *Salmonella enterica *subsp.*enterica *genome sequences were used in this study (Table 1). Genome sequences were downloaded from the Comprehensive Microbial Resource at The Institute for Genomic Research (TIGR; current J. Craig Venter Institute, JCVI) on November 25, 2005. Updated role category information for all genes was obtained from JCVI on October 14, 2008; the *Salmonella *Typhi CT18 genome was used as reference for role categories. While, as of August 20, 2009, 16 fully sequenced *Salmonella *genomes, including the 5 genomes used in our study, were available in GenBank (see Additional file 1), the 5 genomes used were the only fully sequenced *Salmonella *genomes available when our analyses were initiated. These 5 genomes allow for evaluation of evolutionary trends among host-restricted and host adapted *Salmonella *strains as they include the serotypes Typhi, Paratyphi A, and Choleraesuis.

**Table 1:** *Salmonella *genomes used in this study

Serotype	No. of ORFs	Accession No.	Sequencing Center	Reference
Choleraesuis	4801	NC_006905	Chang Gung Univ.	[10]
Paratyphi A	4093	NC_006511	Washington Univ.	[5]
Typhi CT18	4395	NC_003198	Sanger Centre/Imperial College	[54]
Typhi Ty2	4323	NC_004631	Univ. of Wisconsin	[21]
Typhimurium	4553	NC_003197	Washington University Consort.	[53]

### Identification of orthologous genes presents in all five *Salmonella *genomes analyzed

OrthoMCL [28], which has previously been used for prokaryotic genome analyses [20,22], was used to identify orthologous genes in the five *Salmonella *genomes. Orthologs present in all five genomes were aligned using ClustalW [29]. Multiple sequence alignments were carried out on amino acid sequences from each orthologous group, followed by conversion to nucleotide sequence alignments using the PAL2NAL software [30]. This strategy was used to allow for correct alignment of diversified regions in which multiple nucleotide substitution events have taken place; since amino acid sequences are more conserved than DNA sequences, they are easier to align and the final alignments are more reliable. Alignments containing variable sequence lengths or having low alignment scores were manually evaluated and edited, using BioEdit software [31], as previously described [18]. For example, alignments containing sequences with different lengths and alignments that contained multiple indels that caused incorrect alignments were reviewed and edited as detailed in [18].

### Detection of genes under positive selection

**Figure 1:**
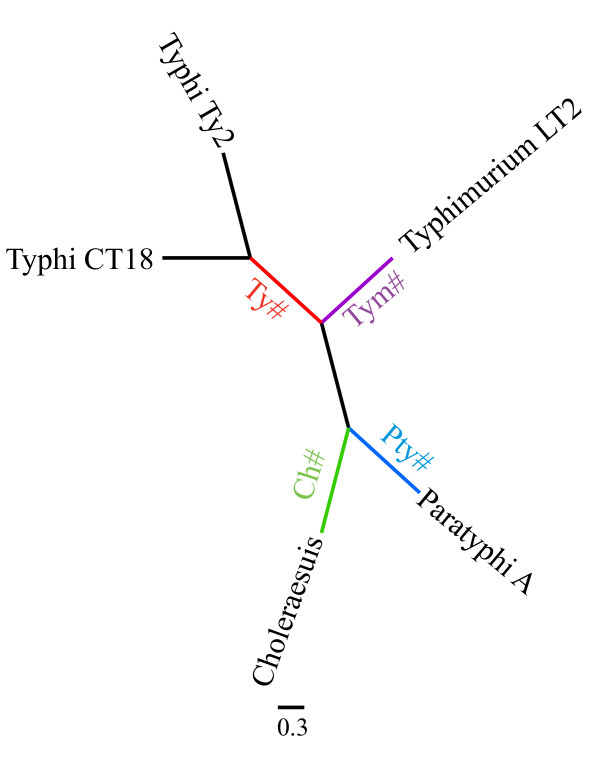
**Example of neighbor joining tree used for positive selection analysis**. Gene specific trees were used for all positive selection analysis. The tree showed here represented the phylogeny of 849 genes. Branches used for branch specific analyses are indicated; Ch# = Choleraesuis branch specific test; Ty# = Typhi branch specific test; Tym# = Typhimurium branch specific test; Pty# = Paratyphi A branch specific test.

Positive selection can be detected by comparing the rate of non-synonymous substitutions (d_N_) to the rate of synonymous substitutions (d_S_). While different methods exist for detection of positive selection, PAML (Phylogenetic Analysis by Maximum Likelihood) was used here as (i) its use for detection of signals of positive selection in bacteria [18,20,23,24,32,33], viruses [34], and eukaryotes [35,36] has been well documented, (ii) it has been shown to have a relatively good power to detect positive selection even with as few as 5 sequences, while keeping the number of false positives low [37], and (iii) it allows for detection of signals of branch specific positive selection. We used two types of tests implemented in PAML v3.15 to identify genes with evidence for positive selection [38], as previously detailed [18]. Briefly, an overall test for positive selection (Test Overall; TO) was carried out to identify genes under positive selection in any or all of the branches of a given phylogeny; this test compares the null model M1a (nearly-neutral) to the alternative model M2a (positive selection) [37]. To identify genes that are under positive selection in specific branches of the *Salmonella *phylogeny, the branch-site test2 [39] was used. The branch-site test was specifically used to identify genes under positive selection in the ancestral branches of (i) the human restricted serotypes Typhi (Ty#) and Paratyphi A (Pty#), (ii) the porcine adapted serotype Choleraesuis (Ch#), and (iii) the unrestricted serotype Typhimurium (Tym#) (Figure 1). Overall, 18 different phylogenetic trees represented the phylogeny of the 3316 *Salmonella *orthologous genes, including one tree that represented the phylogeny of 1198 genes. Both the overall test and the branch site tests were performed using the gene specific trees.

For each test, nested models (one null model that does not allow for positive selection and one alternative model that allows for positive selection) were compared using a Likelihood Ratio Test (LRT) [40]. For each model, three replicates were generated and the maximum likelihood values for each model were used in the LRT in order to eliminate the runs that could not reach the global maximum likelihood score. Tests that yielded LRT values < -0.1 were re-run 10 times and the maximum values for each model were used to calculate the LRT. Negative LRT values (i.e., some tests yielded values ≥ - 0.1) were rounded to zero (*p*-value = 1). For all branch-specific tests, one degree of freedom was used to calculate *p*-values, while for the overall test, two degrees of freedom were used to calculate *p*-values. Because recombination may generate false positive results with PAML, the final analysis of positive selection was carried out only for those genes that showed no evidence for recombination with any of the four methods used to detect evidence of recombination.

### Detection of genes with evidence of recombination

Recombination analyses were performed using GENECONV version 1.81 [41], Maximum χ^2 ^[42], pairwise homoplasy index (PHI) [43] and neighbor similarity score (NSS) [44] to specifically detect evidence of homologous recombination among orthologous genes found in all 5 genomes; the 3316 alignments of orthologous genes used for these analyses thus contained one sequence from each genome and only recombination events between sequences present in the alignment were considered. Except for GENECONV, the approaches used are implemented in PhiPack [43]. GENECONV and Maximum χ^2 ^are substitution distribution methods, while NSS and Phi are compatibility methods [45,46]. None of these tests require that the true phylogenetic tree is known. GENECONV detects the evidence of recombination by assessing the significance of long tracts of identical sites among pairs of sequences in a multiple alignment of informative sites. Maximum χ^2 ^searches for recombination breakpoints in the alignment by comparing the number of polymorphic and non-polymorphic sites downstream and upstream of each putative break point (in this method, all polymorphic sites are initially considered as putative recombination breakpoints). NSS uses pairs of informative sites to detect evidence for recombination by assessing the tendency of neighboring sites to be more compatible than sites that are farther apart. PHI measures the similarity between closely linked sites to assess whether a fragment shows evidence for recombination. GENECONV, Maximum χ^2 ^and NSS were used here as these methods, in a comparison of several methods (not including PHI), were shown to perform best (high power and low false positive rates) for sequences with divergence around 5% - 20% [47], representing a level of divergence expected between different *Salmonella *serotypes. These methods still differ in their relative power and specificity for detecting recombination though (e.g., depending on sequence divergence) and multiple methods were thus used to identify genes with evidence of recombination, particularly to allow for exclusion of any genes that may have evolved through recombination from subsequent positive selection analyses, which may be affected by recombination.

For the GENECONV analyses, the parameter g-scale was set to 1 and inner *p*-values were used to identify genes with evidence for recombination [41]. For Maximum χ^2^, a fixed window size of 2/3 the number of polymorphic sites was used, while for PHI, a window size of 50 nucleotides was used. *P*-values were estimated using 10,000 permutations of the alignment for GENECONV and 1,000 permutations for NSS, Maximum χ^2 ^and PHI.

### Assessment of codon bias, nucleotide diversity and number of informative sites

To assess the codon bias, we identified the effective number of codons used in a gene (N_C_) using the program "chips" in the EMBOSS package [48]. N_C _values range from 20, where one codon is used for each amino acid, to 61, where all alternative synonymous codons are used. Lower values of N_C _indicate higher codon bias in the gene, while higher values of N_C _values indicate lower codon bias. Nucleotide diversity and number of informative sites were obtained from PhiPack outputs.

### Statistical analyses

Correction for multiple testing was performed using the procedure reported by Benjamini and Hochberg [49] as implemented in the program Q-Value [50]. As previously detailed by our group [18], for each *p*-value, the *q*-value was calculated; the *q*-value represents the false discovery rate [FDR], i.e., the expected proportion of false positives among the significant tests. Corrections were performed separately for each test to account for testing of multiple genes. In a preliminary analysis of positive selection, all 3,316 genes were used for FDR correction. As recombination affects the tests for positive selection, the final positive selection analysis was performed using only those 3,046 genes that showed no evidence for recombination; FDR correction for this final positive selection analysis was thus performed with 3,046 genes. As the tests used for positive selection are already conservative [39], a false discovery rate (FDR) cutoff of 20% was used for the positive selection analyses [18]. For recombination analyses, an FDR cut-off of 10% was used to compensate the fact that no correction for multiple tests (GENECONV, NSS, Maximum χ^2 ^and PHI) was carried out due to the high correlation among the tests [18].

Associations between JCVI role categories and number of genes with (i) evidence of positive selection and (ii) evidence of recombination were tested using chi-square tests (or Fisher's exact tests where appropriate). Mann-Whitney U-tests (Wilcoxon tests) were used to determine whether selected continuous variables (i.e., gene length, codon bias, and nucleotide diversity) differed between a given role categories and all other role categories. In addition, Mann-Whitney U-tests were used to test whether the *p*-values of the positive selection tests for genes in a given role category were significantly lower than the *p*-values among the genes in the other role categories. All Mann-Whitney U-tests were performed as one-sided tests. All tests were performed in the Statistical Analysis System (SAS) 9.1 (SAS Institute Inc., Cary, NC).

Bonferroni corrections for all tests were performed based on the number of tests performed. The cut off value for significance was set at 0.05; Bonferroni corrected *p*-values are reported unless otherwise stated. Actual *p*-values are reported unless *p*-values were < 0.001 or < 0.0001.

### Verification of positive selection and recombination patterns in selected genes in a larger *Salmonella *set

For four genes (Table 2), including two genes that showed evidence for positive selection and recombination (i.e., *folK-2*, *sseC*) and two genes that only showed evidence for positive selection (i.e., STM3258, *purE*) in the initial genome wide analyses, gene sequences were determined for an additional 42 *Salmonella *isolates to further test positive selection and recombination patterns. The 42 *Salmonella *isolates were selected to reflect a diversity of human and animal associated serotypes; specifically, the isolates were selected to represent the 15 most common human and animal associated serotypes in the US (as detailed in the 2003 *Salmonella *Annual Report from the US Centers of Disease Control and Prevention [51]) as well as two additional *Salmonella *Typhi isolates. Human and cattle isolates representing the common human and animal associated serotypes were conveniently selected from the strain collection available at Cornell University Food Safety Laboratory, which include human and animal clinical isolates originally obtained from the New York State Department of Health and the Cornell University Animal Health Diagnostic Center, respectively. For common serotypes (e.g., Typhimurium) more isolates were included in this set as compared to less common serotypes (e.g., Dublin) (see Additional file 2 for a listing of all isolates used). Multiple isolates with the same serotype were selected to represent the most common distinct Pulsed Field Gel Electrophoresis (PFGE) and multilocus sequence typing (MLST) types within a given serotype.

**Table 2:** Genes used to confirm positive selection and recombination patterns identified in genome wide analyses

Gene Name	Protein name	JCVI Role Category	Gene length (bp)	Genome analyses results for		Sequence analyses results for^c^	
					
				Positive Selection^a^	Recombination^b^	Positive Selection^d^	Recombination^e^
*folK-2*	2-amino-4-hydroxy-6-hydroxymethyldihydro pteridine pyrophosphokinase	Biosynthesis of cofactors, prosthetic groups, and carriers	480	(*TO, Ty#*)	GEN, MAX	(*Ty#*)	GEN, MAX, NSS
STM3258	Putative PTS system IIA component	Transport and binding proteins	465	Ty#	-	Ty#	-
*sseC*	Probable pathogenicity island effector protein	Unclassified	1455	(*Ch#*)	GEN, MAX	(*TO, Ch#*)	GEN, MAX, NSS, PHI
*purE*	Phosphoribosylamino-imidazole carboxylase, catalytic subunit	Purines, pyrimidines, nucleosides, and nucleotides	510	Ty#	-	Ty#	NSS

^a^positive selection tests that were significant (*Q *< 0.2) are listed; TO = overall test; Ch# = Choleraesuis branch specific test; Ty# = Typhi branch specific test; for genes that showed evidence of recombination, results are shown in a parenthesis as recombination may affect the positive selection analyses.

^b^recombination tests that were significant (*Q *< 0.1) are listed; GEN = GENECONV; MAX = Maximum χ^2^; PHI = pairwise homoplasy; NSS = neighbor similarity score

^c^Results of positive selection and recombination analyses were based on gene sequence data for the 5 genomes and 42 additional *Salmonella *isolates (see Additional file 2); for *folK-2 *and *sseC *sequences were only obtained for 36 additional isolates; for STM3258 sequences were only obtained for 37 additional isolates.

^d^positive selection tests that were significant (*P *< 0.05); for genes that showed evidence of recombination with multiple tests, results are shown in a parenthesis as recombination may affect the positive selection analyses.

^e^recombination tests that were significant (*P *< 0.05)

PCR conditions and primers for *folK-2*, *sseC*, *purE*, and STM3258 amplification are described in Additional file 3. PCR products were purified using Exonuclease I (USB) and shrimp alkaline phosphatase (USB). Purified PCR products were sequenced using the Applied Biosystems Automated 3730 DNA Analyzer at the Cornell University Life Sciences Core Laboratories Center. Big Dye Terminator chemistry and AmpliTaq-FS DNA Polymerase were used for sequencing. Alignments for positive selection and recombination analyses, which were performed as detailed above, were constructed using the gene sequences for the five genomes analyzed and the gene sequences for the additional isolates sequenced.

## Results

### Initial identification and characterization of orthologous genes present in the five *Salmonella *genomes representing serotypes Typhi, Typhimurium, Choleraesuis, and Paratyphi A

Using OrthoMCL, a total of 3323 orthologous genes present in all 5 *Salmonella *genomes were identified. Since seven orthologous genes had low quality alignments, we excluded these genes and used 3316 orthologous genes for the analyses described below. Genes that were not found in all of the five strains were excluded from our analyses. The 3316 core genes represented 69, 81, 73, and 75%, respectively, of the *Salmonella *Choleraesuis, Paratyphi A, Typhimurium, and Typhi genes annotated in the genomes analyzed.

Interestingly, we identified one 2-gene cluster (i.e., STM0947 and STM0948), which was repeated 12 times in the *Salmonella *Choleraesuis genome, present once in Typhimurium genome and absent in the Typhi and Paratyphi A genomes. These two genes encode a putative integrase (STM0947) and a putative cytoplasmic protein (STM0948), which differ by 4 and 1 non-synonymous substitution(s), respectively, between Choleraesuis and Typhimurium LT2. In addition, we identified one other gene (NT03ST2087, encoding a putative Tn10 transposase), which was repeated 7 times in the *Salmonella *Choleraesuis and found once in the *Salmonella *Typhi CT18, while not present in the other genomes analyzed. *Salmonella *Choleraesuis thus appears to contain at least two multi-copy mobile genetic elements.

Genes categorized in the JCVI role categories "Hypothetical Proteins", "Protein synthesis", "Unclassified" and "Unknown function" showed a tendency to have shorter alignments (*P *< 0.001, *P *= 0.027, *P *= 0.002, *P *= 0.017, respectively; one sided U-test) as compared to genes in other role categories, while genes in the JCVI role categories "Amino Acid Biosynthesis", "DNA Metabolism", "Energy Metabolism", and "Transport and Binding Proteins" showed a tendency to have longer alignments (*P *< 0.001, *P *= 0.001, *P *< 0.001, and *P *< 0.001, respectively; one sided U-test) as compared to genes in other role categories.

Genes in the JCVI role categories "Cellular envelope", "Hypothetical proteins", and "Unclassified" showed a tendency to have more non-synonymous substitutions (*P *= 0.009, *P *< 0.001, and *P *< 0.001, respectively; one sided U-test) as compared to genes in other role categories. Genes in the JCVI role categories "Biosynthesis of cofactors, prosthetic groups, and carriers", "Energy Metabolism", and "Transport and Binding Proteins" showed a tendency to have more synonymous substitutions (*P *< 0.001, *P *< 0.001, and *P *= 0.001, respectively; one sided U-test) as compared to genes in other role categories. Genes in the JCVI role categories "Amino acid biosynthesis", "Energy metabolism", "Protein Synthesis", "Purines, pyrimidines, nucleosides, and nucleotides", "Transcription", and "Transport and binding proteins" showed a tendency to have higher codon bias (*P *= 0.006, *P *< 0.001, *P *< 0.001, *P *< 0.001, *P *= 0.033, and *P *= 0.010, respectively; one sided U-test) as compared to genes in other role categories.

### Approximately 8% of core genes show significant evidence for recombination

Among the 3316 orthologous genes, 233 genes showed no substitutions; these genes thus were not analyzed for evidence of homologous recombination (since the methods used cannot detect evidence of recombination if an alignment presents no polymorphisms). While the remaining 3083 genes were analyzed for recombination using GENECONV, only 2849 genes were analyzed using Max χ^2^, NSS and PHI (467 ortholog alignments had ≤1 informative site and thus could not be analyzed with these programs in PhiPack). Overall, 270 genes (8.14% of all 3,316 core genes) showed evidence for recombination in at least one of the four tests used (FDR < 10%). A total of 192, 155, 69, and 20 orthologs showed evidence of recombination using GENECONV, Max χ^2^, NSS and PHI, respectively. Only 10 genes showed evidence for recombination with all 4 approaches (Table 3). Substitution methods (i.e., GENECONV and Maximum χ^2^) thus identified more genes with evidence of recombination as compared to compatibility methods (i.e., NSS and PHI). The differences in the number of genes with evidence of recombination detected with each method are related to (i) the power of the methods to detect recombination in sequences with different divergence and recombination levels, as well as (ii) the number of false positives associated with each method under different scenarios of heterogeneous substitution rates and convergent evolution. For example, GENECONV and Maximum χ^2 ^showed more power to detect recombination as compared to NSS in a study using computer simulations [47], consistent with the observation that both of these methods identified the largest number of genes with evidence of recombination here. Both GENECONV and NSS also have been found, in a study using empirical data, to show higher levels of false positives as compared to Maximum χ^2 ^when the sequences are very divergent [45], while, in another study [43] both NSS and Maximum χ^2 ^have been shown to yield more false positives than PHI particularly in sequences with mutational hot spots. This is consistent with our observation that PHI identified the lowest number of genes with evidence for homologous recombination.

**Table 3:** Genes that show evidence of recombination in all four tests^a^

Gene annotation no. for *S*. Typhimurium LT2	Protein name	Gene name	JCVI Role Category
STM0067	Carbamoyl-phosphate synthase, large subunit	*carB*	Purines, pyrimidines, nucleosides, and nucleotides
STM0224	Surface antigen	*b0177*	Unknown function
STM0540	Conserved hypothetical protein	-	Hypothetical proteins
STM0661	Inosine-uridine preferring nucleoside hydrolase	*iunH*	Purines, pyrimidines, nucleosides, and nucleotides
STM2287	Conserved hypothetical protein	-	Hypothetical proteins
STM2660	ATP-dependent protease, Hsp 100, part of novel	*clpB-1*	Protein fate
STM2947	Sulfite reductase (nADPh) hemoprotein beta-component	-	Central intermediary metabolism
STM2948	Sulfite reductase (nADPh) flavoprotein alpha-component	-	Central intermediary metabolism
STM3174	DNA topoisomerase IV, A subunit	*parC*	DNA metabolism
STM4066	Fructokinase	*cscK*	Energy metabolism

^a^These genes showed evidence for recombination (*Q *< 0.1) in four tests (i.e., GENECONV, Maximum χ^2 ^[Max-χ^2^], pairwise homoplasy index [PHI], and neighbor similarity score [NSS])

**Figure 2:**
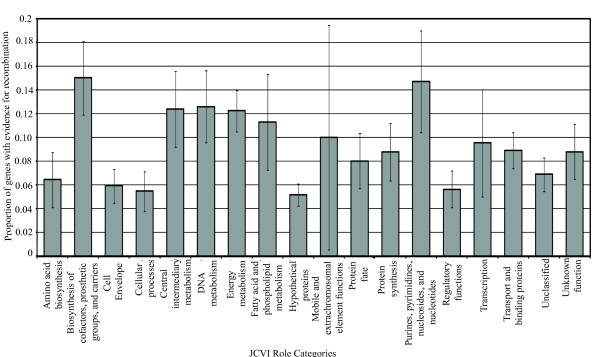
**Proportions of genes with evidence of recombination among individual JCVI role categories**. Genes with evidence for recombination (*Q *< 0.1) in at least one of the four tests were included. Bars indicate estimated standard error for the proportion of genes with evidence of recombination in each role category; standard errors were calculated as square root of p (1-p)/n, where p is the proportion of genes with evidence of positive selection in a given role category, and n is the total number of genes in a given role category. Among the 20 JCVI role categories, two did not include genes with evidence of recombination (i.e., "Signal Transduction" and "Viral functions") and are thus not included in this figure.

When considering all 270 genes identified as having evidence of recombination by at least one method, genes with higher numbers of informative sites (*P *< 0.0001; one sided U-test), longer alignments (*P *< 0.0001; one sided U-test), higher codon bias (*P *< 0.0001; one sided U-test), and higher nucleotide diversity (*P *< 0.0001; one sided U-test) were more likely to have evidence for recombination. An overall chi-square test showed that genes with evidence of recombination were not randomly distributed among the 20 JCVI role categories (*P *< 0.001; Fisher's exact test with Monte Carlo simulation). Subsequent individual chi-square and Fisher's exact tests, determining whether genes with evidence for recombination were associated with individual role categories, showed that genes with evidence of recombination were significantly overrepresented in the role categories "Biosynthesis of cofactors, prosthetic groups, and carriers", "Energy metabolism", "Hypothetical proteins" and "Purines, pyrimidines, nucleosides, and nucleotides" (uncorrected *P *= 0.0035, *P *= 0.0037, *P *= 0.0034, and *P *= 0.0493, respectively) (Figure 2). However, after corrections for multiple comparisons, the associations are not significant (Bonferroni corrected *P *= 0.063, *P *= 0.066, *P *= 0.061, and *P *= 0.887, respectively).

### Initial analysis revealed a total of 81 *Salmonella *genes showing evidence for positive selection

When preliminary positive selection analyses were performed on all 3,316 orthologous genes, 21 genes showed evidence for positive selection (FDR <20%) in the overall test (TO) (Additional file 4). A total of 23, 21, 13, and 14 genes, respectively, showed evidence of positive selection (FDR <20%), using the branch-site test, in the Choleraesuis, Typhi, Typhimurium, and Paratyphi A branch (Additional file 4). As the two Typhi isolates formed a single branch in only the phylogenies for 1261 genes, only these genes were used to test for positive selection in the Typhi branch. While 81 genes showed evidence of positive selection in at least one test (including 11 genes with evidence for positive selection in two tests, see Additional file 4), 32 of these genes also showed evidence of recombination with at least one of the four recombination tests used (Table 4; Additional file 4). Genes with evidence of recombination were more likely to be under positive selection (*P *< 0.0001; Chi-square test). Although this may indicate that positive selection contributes to fixation of new allelic variants that were generated by recombination [18], it may also reflect that the positive selection tests were affected by intragenic recombination [52]. Thus, FDR corrections for positive selection analyses were repeated after removal of the 270 genes with evidence of recombination; these new FDR corrections used 3,046 genes for the overall (TO) test and the branch tests of Choleraesuis, Typhimurium and Paratyphi, and 1,108 genes for the Typhi branch test. All data in the subsequent sections represent the data for genes with no evidence for homologous recombination, unless otherwise stated.

**Table 4:** Evidence of recombination among genes with evidence for positive selection

Test for positive selection^a^	No. of genes with evidence for positive selection and no evidence for recombination	No. of genes with evidence for positive selection that show evidence of recombination with^b^				Total no. of genes with evidence for positive selection and recombination^c^
			
		GENECONV	Max-χ^2^	PHI	NSS	
TO	5	12	7	3	4	13
Ch#	8	11	10	1	3	11
Ty#	16	4	3	0	3	5
Tym#	7	4	4	0	1	4
Pty#	5	8	7	1	2	8

^a^TO = overall test; Ch# = Choleraesuis branch specific test; Ty# = Typhi branch specific test; Tym# = Typhimurium branch specific test; Pty# = Paratyphi A branch specific test

^b^Based on our preliminary analysis, among 3316 orthologous genes, 81 genes showed evidence of positive selection in at least one test. Among 81 genes, 32 genes also showed evidence of recombination with at least one of the four recombination tests used in our study. Statistical analysis showed that genes evidence of recombination were more likely to be under positive selection (*P *< 0.0001; chi-square test). Therefore, we excluded the 270 genes with evidence of recombination from our final positive selection analysis.

^c^This column lists the number of genes that show evidence for recombination and positive selection in a given test (e.g., TO); since many genes showed evidence of recombination in > 1 recombination test, the total number of genes in this column is lower than the sum of the numbers in a given row. While a total of 32 genes showed evidence of recombination and positive selection, the sum of the numbers in this column is > 32 as some genes showed evidence of positive selection in two tests.

### A total of 41 *Salmonella *genes with no evidence of recombination showed evidence of positive selection

Positive selection tests identified 5 genes with evidence for positive selection (FDR <20%) in the overall test (TO) (Table 5). A total of 8, 16, 7, and 5 genes, respectively, showed evidence of positive selection (FDR <20%), using the branch-site test, in the Choleraesuis, Typhi, Typhimurium, and Paratyphi A branches (Table 5; Additional file 5). None of these genes showed of evidence of positive selection in more than one test.

**Table 5:** Genes with evidence for positive selection

Gene annotation no. for *S. *Typhimurium LT2	Gene name	Protein name^a^	Alignment length (bp)	Positive selection (*q*- value)^b^	BEB (*P *>95%)^c^
*Genes in JCVI role category^d ^"Cell Envelope"*					
STM1441	-	Membrane protein, putative	1995	Ch# (0.0043)	-
STM2267	*ompC*	Outer membrane protein C precursor	1134	Ch# (0.0986)	274
STM0743	-	Putative lipoprotein	273	Ch# (0.1830)	-
STM2801	*ygaC*	Conserved hypothetical protein	300	Pty# (0.020)	-
STM0301	*safC*	Outer membrane usher, *Salmonella *atypical fimbria	2508	TO (0.0104)	85, 111, 405, 692
*Genes in JCVI role category "Cellular processes"*					
STM4106	*katG*	Catalase hydroperoxidase HPI(I)	2178	TO (0.0035)	-
STM1425	*ydhE*	Hypothetical integral membrane protein	1371	Tym# (0.0145)	-
*Genes in JCVI role category "Energy metabolism"*					
STM4023	-	Putative 3-hydroxyisobutyrate dehydrogenase	840	Ch# (0.0138)	-
STM3680	*aldB*	Aldehyde dehydrogenase B	1536	Pty# (0.020)	-
STM0698	*pgm*	Phosphoglucomutase, alpha-D-glucose phosphate-specific	1638	Ty# (0.0157)	-
STM3515	*malt*	MalT regulatory protein	2703	Ty# (0.0198)	801
STM4187	*iclR*	Acetate operon repressor	819	Ty# (0.0693)	-
STM0401	*malZ*	Glycosyl hydrolase, family 13	1815	Tym# (0.1005)	-
*Genes in JCVI role category "Hypothetical proteins"*					
STM3329	-	Conserved hypothetical protein TIGR01212	927	Ch# (0.1471)	-
STM1854	-	Hypothetical protein	162	Pty# (0.1973)	32, 40, 44, 45
STM0861	-	Conserved hypothetical protein	471	Tym# (0.1149)	-
STM1515	-	Conserved hypothetical protein TIGR00156 domain protein	384	Ty# (0.0167)	-
STM4015	-	Hypothetical protein	846	Ty# (0.0693)	-
STM4258	-	Conserved hypothetical protein	1386	Ty# (0.0884)	-
STM1532	-	Hypothetical protein	678	Ty# (0.1614)	-
STM1280	-	Conserved hypothetical protein	396	Tym# (0.0145)	-
STM3463	-	Conserved hypothetical protein	201	Tym# (0.1005)	-
*Genes in JCVI role category "Purines, pyrimidines, nucleosides, and nucleotides"*					
STM0534	*purE*	Phosphoribosylaminoimidazole carboxylase, catalytic subunit	507	Ty# (0.0194)	-
STM2806	*nrdI*	NrdI protein	408	Ty# (0.0693)	-
STM2107	*wcaH*	GDP-mannose mannosyl hydrolase	435	Tym# (0.1081)	-
*Genes in JCVI role category "Transport and binding proteins"*					
STM1679	*oppA*	Oligopeptide ABC transporter, periplasmic oligopeptide-binding protein	1605	Ch# (0.0121)	-
STM3685	*mtlA*	PTS system, mannitol-specific IIC component subfamily, putative PTS system IIA component, putative	1914	TO (0.0035)	-
STM3258	-		462	Ty# (0.0157)	124, 139, 143, 144, 147
STM3626	*oppF*	Oligopeptide ABC transporter, ATP-binding protein	1011	Ty# (0.0157)	-
*Genes in JCVI role category "Unclassified and unknown function"*					
STM3592	-	Proton/peptide symporter family protein	1470	TO (0.0104)	-
STM1088	*pipB*	Pathogenicity island encoded protein: SPI5, PipB	873	TO (0.0688)	173
STM0248	-	Histidinol phosphatase-related protein	573	Ch# (< 0.0001)	175, 184, 185, 191
STM3565	-	Acetyltransferase, GNAT family	381	Pty# (0.0391)	-
STM3955	*rarD*	RarD protein	879	Ty# (0.0194)	-
*Genes in JCVI role category "Biosynthesis of cofactors, prosthetic groups, and carriers"*					
STM1450	-	Pyridoxal kinase	666	Ty# (0.0147)	-
STM3057	*ubiH*	2-octaprenyl-6-methoxyphenol hydroxylase, UbiH	1176	Tym# (0.1149)	-
*Genes in JCVI role category "Viral functions"*					
STM2678	*b2611*	Putative membrane protein, CorE	750	Ch# (0.1411)	-
STM4242	-	99% identical to TraF of plasmid R64	1284	Pty# (0.0329)	-
*Genes in other JCVI role categories^e^*					
STM3655	*glyS*	Glycyl-tRNA synthetase, beta subunit	2067	Ty# (0.0393)	313
STM0603	*araT*	Aminotransferase, class I	1158	Ty# (0.1976)	-
STM0395	-	Exonuclease SbcC, putative	3096	Ty# (0.1041)	-

^a^Protein designations were taken from the Typhi CT18 annotation; where limited annotation information was available, additional information was extracted from JCVI primary annotations and Typhimurium LT2 and Paratyphi A annotations

^b^tests that were significant for positive selection (FDR <20%) are shown; TO = overall test; Ch# = Choleraesuis branch specific test; Pty# = Paratyphi A branch specific test; Ty# = Typhi branch specific test; Tym# = Typhimurium branch specific test; numbers in brackets indicate *q*-values

^c^aa sites identified by Bayes Empirical Bayes (BEB) as having probability > 95% of being under positive selection are shown; aa sites are based on site location in the alignment (alignments for genes under positive selection are provided as Additional file 6)

^d^Role categories were assigned based on annotations for *S*. Typhi CT18; JCVI locus names for Typhi CT18 for these genes are listed in Additional file 5.

^e^Other role categories include Protein synthesis (STM3655), Central intermediary metabolism (STM0603), DNA metabolism (STM0395)

**Figure 3:**
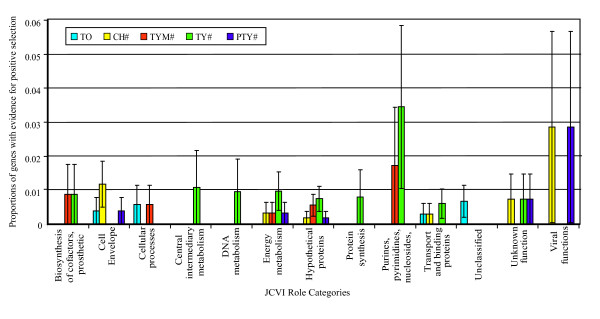
**Proportions of genes with evidence of positive selection among individual JCVI role categories**. Only genes that showed no evidence for recombination were used to generate the data showed here. Bars indicate estimated standard error for the proportion of genes with evidence of positive selection in each role category; standard errors were calculated as the square root of p (1-p)/n, where p is the frequency of genes with evidence of positive selection in a given role category, and n is the total number of genes in a given role category. Among the 20 JCVI role categories, seven did not include genes with evidence of positive selection and are thus not included in this figure. For each role category, proportion of genes with evidence of positive selection in the overall test (TO) and each of the four branch specific tests (Ch# = Choleraesuis branch specific test; Ty# = Typhi branch specific test; Tym# = Typhimurium branch specific test; Pty# = Paratyphi A branch specific test) are shown.

No association between the low effective number of codons used by a gene (Nc) and evidence for positive selection was observed (*P *> 0.05; one-sided U-test) suggesting that results of positive selection analyses were not biased by constrains on codon usage, which could result in a low synonymous substitution rate in these genes. Moreover, no association between low d_S _(the number of synonymous substitutions divided by the number of synonymous sites) and positively selected genes was observed (*P *> 0.05; one-sided, U-test), supporting that the results were not biased by a low synonymous substitution rate. A Fisher's exact test did not find any significant overall association between the 20 JCVI role categories and the genes under positive selection (Figure 3), possibly due to the low number of genes under positive selection in each role category. To further test for associations between positive selection and gene role category, we thus assessed, for each of the role categories, whether the distribution of the *p*-values for each positive selection test deviated from the random distribution, using the non-parametric U-test. The JCVI role category "Hypothetical proteins" showed significant trends of having genes with low *p*-values in the Choleraesuis, Typhimurium and Paratyphi A branch specific tests for positive selection (Bonferroni corrected *P *= 0.042, *P *= 0.034 and *P *< 0.001, respectively; one sided U-test) as compared to genes in other role categories. In addition, genes in the JCVI role categories "Unclassified" and "Protein synthesis" showed a significant trend of having low *p*-values in the Choleraesuis and Typhimurium branch tests for positive selection, respectively, as compared to genes in other role categories (Bonferroni corrected *P *= 0.002 and *P *= 0.013, respectively; one sided U-test).

Among *Salmonella *pathogenicity islands 1 through 6, three genes showed evidence for positive selection (i.e., *pipB*, STM1088 [*siiB*], and *safC*; see Table 6). Overall, 102 of the orthologs analyzed were located in the 6 *Salmonella *pathogenicity islands [53,54]; genes in the pathogenicity islands were not significantly overrepresented (*P *> 0.05; Fisher's exact test) among the genes with evidence for positive selection. In addition, three SPI-1 genes (i.e., *spaM, iagB*, and *sipD*), and one SPI-2 gene (*ssaI*) showed uncorrected *p*-values < 0.05 in the TO positive selection test (*P *= 0.049, 0.017, 0.003 and 0.047, respectively), but failed to meet the FDR cutoff (*q*-values = 1, 1, 0.925, and 1, respectively). Similarly, one SPI-2 gene (*sseF*) showed a low uncorrected *p*-value (*P *= 0.001) in the Choleraesuis branch test, but failed to meet the FDR cutoff (*q*-value = 0.332).

**Table 6:** *Salmonella *pathogenicity island (SPI) genes with evidence of positive selection and recombination

SPIs^a^	Location^b^	No. of orthologous genes found in SPI	No. of genes with evidence for positive selection	No. of genes with evidence for recombination
1	STM2865-2914	34	0	1 (*stpA*)
2	STM1379-1422	32	0	3 (*sseC, sseD*, STM1379)
3	STM3752-3764 STM3766-3775	5	0	0
4	STM4257-4262	7	1 (*siiB*)	0
5	STM1087-1094	4	1 (*pipB*)	0
6	NT03ST0297-0356	20	1 (*safC*)	4 (*sciK, sciG*, STM0289, STM0296)

^a^This table lists genes in the *Salmonella *Pathogenicity islands (SPIs) 1 to 6

^b^Genes in SPIs 1 to 5 are reported as described by [53] using primary annotation locus numbers for *Salmonella *Typhimurium LT2; genes in SPI-6 are reported as described by [54] using JCVI locus numbers for *Salmonella *Typhi CT18

Interestingly, *ompC *showed evidence for positive selection in our study (Table 5) as well as in a previous study of *Shigella *and *E. coli *[24]. Our analyses showed that aa residues 228 and 274 show evidence for positive selection (Additional file 6), while aa 163, 202, and 203 showed evidence for positive selection in *E. coli *and *Shigella *[24]. *Salmonella *OmpC aa site 228, which was found to be under positive selection here, is located in a region that is absent from the *E. coli *and present in *Shigella *OmpC, while *Salmonella *OmpC aa site 274 is located in a region that is absent from OmpC in both *E. coli *and *Shigella*.

### Verification of positive selection and recombination patterns, identified by genome wide analyses, for four genes among 42 *Salmonella *isolates

In order to confirm positive selection and recombination patterns identified by the full genome analyses, we used a larger set of 42 *Salmonella *isolates to sequence and analyze four genes, including two genes that showed evidence for positive selection and recombination (i.e., *folK-2*, *sseC*) and two genes that only showed evidence for positive selection (i.e., STM3258, *purE*). *folK-2*, which encodes an enzyme involved in the synthesis of folic acid, could not be PCR amplified in 6 *Salmonella *isolates, representing serotypes Montevideo (n = 2), Oranienburg, Javiana, Urbana, and Muenster. Analyses of 41 *folK-2 *sequences (5 sequences from the genomes and 36 newly determined sequences) confirmed that this gene shows evidence for recombination (Table 2).*sseC*, which is located in the *Salmonella *pathogenicity island 2, could not be PCR amplified in 6 *Salmonella *isolates, representing serotypes Agona (n = 2), Havana, Kentucky, and Mbandaka (n = 2). Analyses of the *sseC *sequences also confirmed that this gene shows evidence for recombination (Table 2). The STM3258 gene, which encodes a putative PTS component, could not be PCR amplified in one *Salmonella *Typhimurium and three serotype 4,5,12:i:-isolates. Results from the analyses of the resulting 43 STM3258 gene sequences was consistent with the genome analyses data and confirmed that this gene shows no evidence for recombination, but is under positive selection in the *Salmonella *Typhi branch. *purE*, which encodes an enzyme involved in the synthesis of purine ribonucleotide, was successfully amplified and sequenced in all 42 isolates; analyses of the resulting sequences also found evidence for positive selection in the *Salmonella *Typhi branch (Table 2); one test (NSS) on all 47 *purE *gene sequences found evidence for recombination in this gene (*P *< 0.001).

## Discussion

In this study, we used 5 *Salmonella *genomes representing host restricted (i.e., Typhi and Paratyphi A), host adapted (i.e., Choleraesuis), and unrestricted (i.e., Typhimurium) serotypes to study the evolution of core genes in different *Salmonella *serotypes. A total of 3,316 orthologs found in these 5 *Salmonella *genomes were used to (i) identify genes with evidence of recombination and (ii) identify genes under positive selection. Positive selection and recombination patterns for four genes of interest were confirmed in a larger set of isolates representing 23 different serotypes. Overall, our data show that, among the serotypes evaluated, (i) less than 10% of *Salmonella *genes in the core genome show evidence for homologous recombination, (ii) a number of core *Salmonella *genes are under positive selection, including genes that appear to contribute to virulence, and (iii) the cell surface protein *ompC*, which may contribute to multi drug resistance in *Salmonella*, is targeted by positive selection in both *Salmonella *and *E. coli *[24].

### Less than 10% of *Salmonella *genes show evidence for intragenic recombination

Since the first bacterial genome was sequenced in 1995, comparative tools have shown that horizontal gene transfer is the major process for the evolution of prokaryotes [12,14,55]. Horizontal gene transfer has also been proposed to have played an important role in the evolution of the *Salmonella *genome. *Salmonella *Typhimurium LT2 seems to have acquired a number of novel genomic regions after the divergence from *E. coli *around 100 millions years ago [56] and it has been estimated that 25% of the *Salmonella *Typhimurium genome may have been introduced by horizontal gene transfer [3]. Groups of genes introduced by horizontal gene transfer include prophages and *Salmonella *pathogenicity islands (SPIs) [13]. While the role of horizontal gene transfer in introducing novel genes into the *Salmonella *genome has been well established, our analyses show that horizontal transfer (and recombination) of homologous genes also plays an important role in the diversification of *Salmonella*; 270 of the 3316 genes characterized (8.1%) showed evidence for intragenic homologous recombination. By comparison, analysis of four *E. coli *and two *Shigella *genomes found 236 genes with evidence for intragenic recombination, representing approximately 6.3% of genes analyzed [24]. Chen et al. [23] reported that 12.8% of core genome genes, found in seven *E. coli *genomes, showed evidence for recombination. A study of 410 genes present in six *E. coli *and six *Salmonella enterica *genomes reported that 23% of these genes showed evidence of recombination in *Salmonella*; this estimate may be higher than the one reported here as the 410 genes evaluated do not represent a random sample of the *Salmonella *core genome [25]. Interestingly, even novel genes that were initially introduced into the *Salmonella *genome through horizontal gene transfer and non-homologous recombination, showed evidence for further subsequent diversification through homologous recombination (e.g., one and two genes in SPI-1 and 2, respectively, showed evidence for intragenic recombination). A recent analysis by Didelot et al. [57] also suggested that convergence of *Salmonella *Typhi and Paratyphi A, two human host-restricted serotypes, through >100 recombination events involving both transfer of novel genes as well as transfer of homologous genes, further supporting the importance of horizontal transfer of homologous gene sequences in the evolution of *Salmonella *[53].

### A number of core *Salmonella *genes are under positive selection, including genes that appear to contribute to virulence and systemic infection

A total of 1.2% of genes found in all five *Salmonella *genomes (i.e., 41 genes) showed evidence for positive selection and no evidence for recombination. While 5 genes showed evidence for positive selection in the overall analyses, 36 genes showed evidence for positive selection only in specific branches, indicating considerable branch specific positive selection in the *Salmonella *serotypes evaluated. Previously, Petersen et al. [24] reported that, among 3,505 *E. coli *and *Shigella *genes that showed no evidence for recombination, a total of 23 genes (0.66%) showed evidence for positive selection. Among Gram-positive pathogens, Orsi et al. [18] reported that 36 *L. monocytogenes *and *L. innocua *genes (1.6%) showed evidence of positive selection (among a total of 2267 genes analyzed), while Lefebure and Stanhope [20] reported that 11 to 34% of the genes in the *Streptococcus *core genome showed evidence for positive selection, although this study did not control for multiple comparisons and thus may have somewhat overestimated the number of genes under positive selection. Recently, Lefebure and Stanhope [22] showed that 92.5% of non-recombinant core genome loci are under positive selection, in at least one lineage, in 17 *Campylobacter *genomes, which represented 8 different species. While, these different analyses suggest that the proportion of genes with evidence for positive selection appears to vary considerably between different bacterial species or genera, methodological aspects (e.g., approaches used to correct for multiple comparisons, approaches used to identify genes with evidence for recombination) may also affect the number of genes identified as showing evidence for positive selection.

Interestingly, three *Salmonella *genes with evidence for positive selection were located in *Salmonella *pathogenicity islands (SPIs). SPIs are chromosomal regions that contain genes contributing to a particular virulence phenotype [26,58,59]. So far, five common SPIs (i.e., SPI-1 through SPI-5), found among the majority of *Salmonella enterica *strains, as well as a number of additional less common SPIs have been reported. *siiB*, which showed evidence for positive selection, is located in SPI-4 and encodes a probable membrane protein (putative methyl-accepting chemotaxis protein). Morgan et al. [60] reported that the SPI-4 genes *siiD, siiE*, and *siiF *play a role in *Salmonella *Typhimurium intestinal colonization of calves. Kiss et al. [61] specifically showed that a *Salmonella *Typhimurium strain lacking *siiB *shows reduced secretion of SiiE, as compared to the wildtype, suggesting a possible involvement of *siiB *in calf virulence (as an *siiE *mutant showed reduced colonization in a calf model [60]). *pipB*, located in SPI-5, also showed evidence for positive selection. SPI-5 encodes T3SS-1 and T3SS-2 effector proteins [62]. PipB localizes to the *Salmonella *Containing Vacuole (SCV) in mammalian host cells [63]. In addition, Wood et al. [62] reported that a *pipB *null mutant showed reduced intestinal secretory and inflammatory responses in ligated bovine ileal loops, suggesting that this, as well as other genes in SPI-5, may contribute to bovine enteric infections. PipB also appears to be required for colonization of the cecum, by *Salmonella *Typhimurium, in chickens [64]. *safC*, a gene located in SPI-6 [54], a region called *Salmonella enterica *centrisome 7 genomic island (SCI) in *Salmonella *Typhimurium [65], was also found to be under positive selection. *safC *encodes an outer membrane usher protein for *Salmonella *atypical fimbriae [65]. While a *Salmonella *Typhimurium strain with a deletion of SPI-6 showed reduced ability to invade Hep2 cells [65], we are not aware of any studies characterizing virulence of a *safC *null mutant. While the SPI-2 genes *sseC *and *sseF *have previously been reported to (i) show evidence for differential evolution [66] and (ii) contain distinct clusters of polymorphic sites that might be unique to the human adapted serotypes Typhi and Paratyphi [67], these genes did not show evidence for positive selection in our final analyses. Both *sseC *and *sseF *showed evidence for positive selection in the Choleraesuis branch in our initial analysis, but *sseC *was removed from the final analysis as this gene also showed evidence of recombination and *sseF *did not meet the 20% cutoff for FDR. In combination with a previous study [66] that reported that a number of genes located in *Salmonella *pathogenicity islands show evidence for differential evolution in different *Salmonella *serotypes, our findings do support that positive selection contributes to evolution of pathogenicity island genes in *Salmonella*, even though further analyses on larger data sets will be needed to clarify the contributions of positive selection and recombination to evolution of these genes.

Overall, three genes in the JCVI role category "Purine, pyrimidine, nucleoside and nucleotide biosynthesis" (i.e., *wcaH, purE *and *nrdI*) showed evidence for positive selection (while showing no evidence for recombination). *wcaH*, which encodes a GDP-mannose mannosyl hydrolase, is under positive selection in the Typhimurium branch, while *purE *and *nrdI *were found to be under positive selection in the Typhi branch. *purE *encodes a phosphoribosylaminoimidazole carboxylase, while *nrdI*, which is located in an operon with genes that encode a Class 1b ribonucleotide reductase, encodes a small flavoprotein with unknown function in *Streptococcus pyogenes *[68]. Positive selection for *purE *in the *Salmonella *Typhi branch was also confirmed in our analyses of 22 human and 20 animal *Salmonella *isolates, which included two additional Typhi strains. This is a striking finding since Samant et al. [69] recently reported that *de novo *nucleotide biosynthesis is essential for bacterial growth in blood. As *Salmonella *Typhi predominantly causes systemic septicemic infections in humans, these findings suggest that adaptive changes in genes encoding purine, pyrimidine, nucleoside and nucleotide biosynthesis functions may have been critical in the evolution of this host restricted human pathogen. Our findings thus further support that development of novel drugs targeting appropriate purine, pyrimidine, nucleoside and nucleotide biosynthesis pathways may represent an opportunity for therapeutic approaches for bacterial pathogens causing septicemic infections [69].

Additional genes with evidence for positive selection and possible roles in host infection include *katG*, which encodes a catalase. While antioxidant defenses mechanism appear to contribute to virulence in a number of pathogens, *Salmonella katG *null mutations have shown no affect on *Salmonella*'s ability to survive inside phagocytic cells and in a murine model of infection [70]. The importance of adaptive changes in *Salmonella katG *thus remains to be determined. It seems possible that adaptive changes in genes involved in anaerobic growth may contribute to an improved ability of different strains of this gastrointestinal pathogen to survive under anaerobic conditions encountered in the intestinal tract. We also identified a number of genes with evidence for positive selection that have no apparent link to infection and virulence, including *malZ*, *malT*, and *mtlA*, which encode, respectively, a maltodextrin glucosidase, a transcriptional activator of *mal *genes, and a mannitol specific PTS system component. While it has been proposed that horizontal transfer of genes encoding proteins involved in acquisition and synthesis of nutrients and genes encoding components of metabolic networks is critical as bacteria adapt to specific environments and ecological niches [12], our findings suggest that positive selection of genes encoding metabolic capabilities also contribute to adaptation to new environments.

### Cell surface proteins are targeted by positive selection in both *Salmonella *and *E. coli*

While we identified, in our preliminary analysis, three genes encoding outer membrane proteins (*ompC, ompS1 *and *ompS2*) that showed evidence for positive selection, only *ompC *showed no evidence of recombination. *ompC*, a highly expressed *omp *gene, encodes a protein that not only appears to play a role in *Salmonella *virulence [71], but also is a receptor for Gifsy-1 and Gifsy-2 phages [72]. An analysis of six *E. coli *and *Shigella *genomes also found that three *omp *genes (i.e., *ompF, ompC *and *ompA*) showed evidence of positive selection [24], while Chen et al. [23] reported that *ompC *and *ompF *were under positive selection in uropathogenic *E. coli *strains. Furthermore, genes encoding the outer membrane proteins OmpA and OmpB showed evidence for positive selection in *Rickettsia *spp. [73]. Overall, these data strongly suggest that adaptive changes in genes encoding outer membrane proteins critically contribute to the evolution of a variety of bacteria, including pathogenic enterobacteriaciae. In particular, *ompC*, which encodes one of the most abundant *E. coli *proteins [24], appears to be under positive selection in a number of pathogenic enterobacteriaciae. As proposed by Petersen et al. [24], positive selection in *omp *genes may be an important mechanism that facilitates adaptation of bacterial pathogens allowing them to escape recognition by the host immune system and phages. In addition, mutations in porin genes (e.g., those belonging to OmpC and OmpF groups), as well as changes in Omp expression levels, have been linked to increased resistance to β-lactam antibiotics [74-76]. For example, under strong antibiotic pressure, bacteria can reduce the influx of antibiotic through downregulation of porin expression or expression of modified porins. Positive selection in porin genes, particularly *ompC *thus may also be associated with selection to increase antibiotic resistance. These findings provide potentially interesting avenues for future mutagenesis studies to elucidate the role of *ompC *polymorphisms in various phenotypes, including β-lactam resistance.

## Conclusion

Our analyses strongly suggest that both homologous recombination and positive selection (particularly lineage specific positive selection) contribute to the evolution of the *Salmonella *core genome, at least in the serotypes analyzed here. While genes with evidence of positive selection identified here may provide promising targets for future mutational studies aimed at further identifying mechanisms that contribute to *Salmonella *diversification, including its adaptation to specific host species, one cannot extrapolate our findings on a few *Salmonella *serotypes to other serotypes unless additional analyses are performed. The relevance of the lineage specific positive selection patterns identified here is supported, though, by the convergence of the positive selection patterns identified in the *Salmonella *Typhi lineage (i.e., for genes encoding proteins involved in purine, pyrimidine, nucleoside and nucleotide biosynthesis) and experimental evidence that genes involved in *de novo *nucleotide biosynthesis are essential for bacterial growth in blood [69].

In conjunction with previous genome wide studies on positive selection in uropathogenic *E. coli *[23], *Shigella *and *E. coli *[24], *Listeria *spp. [18], *Campylobacter *[22] and *Streptococcus *spp. [20], our data clearly indicate the positive selection and homologous recombination among core genome genes play an important role in the evolution of bacterial pathogens, in addition to the well established importance of gene acquisition and deletion. Positive selection and homologous recombination also appear to contribute to further evolution of novel genes initially acquired by lateral gene transfer, such as selected genes in the *Salmonella *pathogenicity islands. As additional pathogen genomes, including additional *Salmonella *genomes, have and continue to become available, positive selection and recombination analyses on larger numbers of genomes will further improve our understanding of bacterial pathogens.

## List of abbreviations used

TO: Test Overall; an overall test for positive selection, which was carried out using the null model M1a (Nearly-neutral) and the alternative model M2a in PAML; Ch#: the Choleraesuis branch specific test for positive selection, which was carried out in PAML using the branch-site test2 and the porcine adapted serotype Choleraesuis branch; Ty#: the Typhi branch specific test for positive selection, which was carried out in PAML using the branch-site test2 and the human restricted serotypes Typhi branch; Tym#: the Typhimurium branch specific test for positive selection, which was carried out in PAML using the branch-site test2 and the unrestricted serotype Typhimurium branch; Pty#: the Paratyphi A branch specific test for positive selection, which was carried out in PAML using the branch-site test2 and the human restricted serotypes Paratyphi A; GENECONV: Statistical Test for Detecting Gene Conversion; this test for evidence of recombination was performed using GENECONV version 1.81; Max χ^2^: Maximum χ^2^; this test for evidence of recombination was performed using Maximum χ^2 ^implemented in the PhiPack software package; NSS: Neighbor Similarity Score; this test for evidence of recombination was performed using Neighbor Similarity Score implemented in the PhiPack software package; PHI: Pairwise Homoplasy Index; this test for evidence of recombination was performed using Pairwise Homoplasy Index implemented in the PhiPack software package.

## Authors' contributions

YS performed, and interpreted the phylogenetic and statistical analyses, performed some sequencing experiments, and drafted the manuscript. RHO outlined the phylogenetic and statistical analyses and helped with their performance and interpretation as well with drafting the manuscript. LR performed some sequencing experiments. QS performed orthologous gene clustering and alignment, and implemented the analysis on the parallel computer cluster. MW supervised the project, participated in the design of the study and data interpretation, and finalized the manuscript. All authors read and approved the final manuscript.

## Supplementary Material

Additional file 1 Additional completed Salmonella genomes available in Genbank as of 08/20/2009, but not used in our study. Click here for file

Additional file 2 ***Salmonella *****isolates (n = 42) used to verify genome wide positive selection and recombination patterns in four selected genes.** Click here for file

Additional file 3 PCR conditions and primers for the four genes that were used to verify genome wide positive selection and recombination patterns in an additional 42 *Salmonella *isolates. Click here for file

Additional file 4 Detailed information for 81 genes showing evidence for positive selection from initial results for positive selection analysis (performed using all genes, including those with evidence of recombination). Click here for file

Additional file 5 Detailed information for 41 genes showing evidence for positive selection from positive selection analysis for genes without recombination. Click here for file

Additional file 6 Alignments for genes under positive selection. Click here for file

## References

[B1] Brenner FW Villar RG Angulo FJ Tauxe R Swaminathan B *Salmonella *nomenclature - Guest commentary J Clin Microbiol 2000 38 7 2465 2467 10878026 10.1128/jcm.38.7.2465-2467.2000 PMC86943

[B2] Uzzau S Brown DJ Wallis T Rubino S Leori G Bernard S Casadesus J Platt DJ Olsen JE Host adapted serotypes of *Salmonella enterica* Epidemiol Infect 2000 125 2 229 255 10.1017/S0950268899004379 11117946 PMC2869595

[B3] Kidgell C Reichard U Wain J Linz B Torpdahl M Dougan G Achtman M *Salmonella *typhi, the causative agent of typhoid fever, is approximately 50,000 years old Infect Genet Evol 2002 2 1 39 45 10.1016/S1567-1348(02)00089-8 12797999

[B4] Baker S Dougan G The Genome of *Salmonella enterica *Serovar Typhi Clin Infect Dis 2007 45 Suppl 1 S29 S33 10.1086/518143 17582565

[B5] McClelland M Sanderson KE Clifton SW Latreille P Porwollik S Sabo A Meyer R Bieri T Ozersky P McLellan M Harkins CR Wang CY Nguyen C Berghoff A Elliott G Kohlberg S Strong C Du FY Carter J Kremizki C Layman D Leonard S Sun H Fulton L Nash W Miner T Minx P Delehaunty K Fronick C Magrini V Nhan M Warren W Florea L Spieth J Wilson RK Comparison of genome degradation in Paratyphi A and Typhi, human-restricted serovars of *Salmonella enterica *that cause typhoid Nat Gen 2004 36 12 1268 1274 10.1038/ng1470 15531882

[B6] Ross IL Heuzenroeder MW Discrimination within phenotypically closely related definitive types of *Salmonella enterica *serovar typhimurium by the multiple amplification of phage locus typing technique J Clin Microbiol 2005 43 4 1604 1611 10.1128/JCM.43.4.1604-1611.2005 15814973 PMC1081374

[B7] Langvad B Skov MN Rattenborg E Olsen JE Baggesen DL Transmission routes of *Salmonella *Typhimurium DT 104 between 14 cattle and pig herds in Denmark demonstrated by molecular fingerprinting J Appl Microbiol 2006 101 4 883 890 10.1111/j.1365-2672.2006.02992.x 16968300

[B8] Nagano N Oana S Nagano Y Arakawa Y A severe *Salmonella enterica *serotype Paratyphi B infection in a child related to a pet turtle, Trachemys scripta elegans Jpn J Infect Dis 2006 59 2 132 134 16632917

[B9] Swaminathan B Gerner-Smidt P Foodborne disease trends and reports Foodborne Pathog Dis 2006 3 3 220 221 10.1089/fpd.2006.3.220 16972769

[B10] Chiu CH Tang P Chu CS Hu SN Bao QY Yu J Chou YY Wang HS Lee YS The genome sequence of *Salmonella enterica *serovar Choleraesuis, a highly invasive and resistant zoonotic pathogen Nucleic Acids Res 2005 33 5 1690 1698 10.1093/nar/gki297 15781495 PMC1069006

[B11] Gal-Mor O Valdez Y Finlay BB The temperature-sensing protein TlpA is repressed by PhoP and dispensable for virulence of *Salmonella enterica *serovar Typhimurium in mice Microb Infect 2006 8 8 2154 2162 10.1016/j.micinf.2006.04.015 16782389

[B12] Pal C Papp B Lercher MJ Adaptive evolution of bacterial metabolic networks by horizontal gene transfer Nat Genet 2005 37 12 1372 1375 10.1038/ng1686 16311593

[B13] Porwollik S McClelland M Lateral gene transfer in *Salmonella* Microbes Infect 2003 5 11 977 989 10.1016/S1286-4579(03)00186-2 12941390

[B14] Lercher MJ Pal C Integration of horizontally transferred genes into regulatory interaction networks takes many million years Mol Biol Evol 2008 25 3 559 567 10.1093/molbev/msm283 18158322

[B15] Schmidt H Hensel M Pathogenicity islands in bacterial pathogenesis Clin Microbiol Rev 2004 17 1 14 56 10.1128/CMR.17.1.14-56.2004 14726454 PMC321463

[B16] Thomson NR Clayton DJ Windhorst D Vernikos G Davidson S Churcher C Quail MA Stevens M Jones MA Watson M Barron A Layton A Pickard D Kingsley RA Bignell A Clark L Harris B Ormond D Abdellah Z Brooks K Cherevach I Chillingworth T Woodward J Norberczak H Lord A Arrowsmith C Jagels K Moule S Mungall K Sanders M Whitehead S Chabalgoity JA Maskell D Humphrey T Roberts M Barrow PA Dougan G Parkhill J Comparative genome analysis of *Salmonella *Enteritidis PT4 and *Salmonella *Gallinarum 287/91 provides insights into evolutionary and host adaptation pathways Genome Res 2008 18 10 1624 1637 10.1101/gr.077404.108 18583645 PMC2556274

[B17] Porwollik S Boyd EF Choy C Cheng P Florea L Proctor E McClelland M Characterization of *Salmonella enterica *subspecies I genovars by use of microarrays J Bacteriol 2004 186 17 5883 5898 10.1128/JB.186.17.5883-5898.2004 15317794 PMC516822

[B18] Orsi RH Sun Q Wiedmann M Genome-wide analyses reveal lineage specific contributions of positive selection and recombination to the evolution of *Listeria monocytogenes* BMC Evol Biol 2008 8 233 254 10.1186/1471-2148-8-233 18700032 PMC2532693

[B19] Chen ZH Schneider TD Comparative analysis of tandem T7-like promoter containing regions in enterobacterial genomes reveals a novel group of genetic islands Nucleic Acids Res 2006 34 4 1133 1147 10.1093/nar/gkj511 16493139 PMC1380254

[B20] Lefebure T Stanhope MJ Evolution of the core and pan-genome of *Streptococcus*: positive selection, recombination, and genome composition Genome Biol 2007 8 5 R71 10.1186/gb-2007-8-5-r71 17475002 PMC1929146

[B21] Deng W Liou SR Plunkett G Mayhew GF Rose DJ Burland V Kodoyianni V Schwartz DC Blattner FR Comparative genomics of *Salmonella enterica *serovar typhi strains Ty2 and CT18 J Bacteriol 2003 185 7 2330 2337 10.1128/JB.185.7.2330-2337.2003 12644504 PMC151493

[B22] Lefebure T Stanhope MJ Pervasive, genome-wide positive selection leading to functional divergence in the bacterial genus *Campylobacter* Genome Res 2009 19 7 1224 1232 10.1101/gr.089250.108 19304960 PMC2704436

[B23] Chen SL Hung CS Xu J Reigstad CS Magrini V Sabo A Blasiar D Bieri T Meyer RR Ozersky P Armstrong JR Fulton RS Latreille JP Spieth J Hooton TM Mardis ER Hultgren SJ Gordon JI Identification of genes subject to positive selection in uropathogenic strains of *Escherichia coli*: a comparative genomics approach Proc Natl Acad Sci USA 2006 103 15 5977 5982 10.1073/pnas.0600938103 16585510 PMC1424661

[B24] Petersen L Bollback JP Dimmic M Hubisz M Nielsen R Genes under positive selection in *Escherichia coli* Genome Res 2007 17 9 1336 1343 10.1101/gr.6254707 17675366 PMC1950902

[B25] Charlesworth J Eyre-Walker A The rate of adaptive evolution in enteric bacteria Mol Biol Evol 2006 23 7 1348 1356 10.1093/molbev/msk025 16621913

[B26] Marcus SL Brumell JH Pfeifer CG Finlay BB *Salmonella *pathogenicity islands: big virulence in small packages Microb Infect 2000 2 2 145 156 10.1016/S1286-4579(00)00273-2 10742687

[B27] Kelly BG Vespermann A Bolton DJ The role of horizontal gene transfer in the evolution of selected foodborne bacterial pathogens Food Chem Toxicol 2009 47 5 951 968 10.1016/j.fct.2008.02.006 18420329

[B28] Li L Stoeckert CJ Jr Roos DS OrthoMCL: identification of ortholog groups for eukaryotic genomes Genome Res 2003 13 9 2178 2189 10.1101/gr.1224503 12952885 PMC403725

[B29] Thompson JD Higgins DG Gibson TJ CLUSTAL W: improving the sensitivity of progressive multiple sequence alignment through sequence weighting, position-specific gap penalties and weight matrix choice Nucleic Acids Res 1994 22 22 4673 4680 10.1093/nar/22.22.4673 7984417 PMC308517

[B30] Suyama M Torrents D Bork P PAL2NAL: robust conversion of protein sequence alignments into the corresponding codon alignments Nucleic Acids Res 2006 34 Web Server W609 612 10.1093/nar/gkl315 16845082 PMC1538804

[B31] Hall TA BioEdit: a user-friendly biological sequence alignment editor and analysis program for Windows 95/98/NT Nucl Acids Symp Ser 1999 41 95 98

[B32] Urwin R Holmes EC Fox AJ Derrick JP Maiden MC Phylogenetic evidence for frequent positive selection and recombination in the meningococcal surface antigen PorB Mol Biol Evol 2002 19 10 1686 1694 12270895 10.1093/oxfordjournals.molbev.a003991

[B33] Andrews TD Gojobori T Strong positive selection and recombination drive the antigenic variation of the PilE protein of the human pathogen *Neisseria meningitidis* Genetics 2004 166 1 25 32 10.1534/genetics.166.1.25 15020403 PMC1470718

[B34] Twiddy SS Woelk CH Holmes EC Phylogenetic evidence for adaptive evolution of dengue viruses in nature J Gen Virol 2002 83 Pt 7 1679 1689 12075087 10.1099/0022-1317-83-7-1679

[B35] Nielsen R Bustamante C Clark AG Glanowski S Sackton TB Hubisz MJ Fledel-Alon A Tanenbaum DM Civello D White TJ Sninsky J Adams MD Cargill M A scan for positively selected genes in the genomes of humans and chimpanzees PLoS Biol 2005 3 6 e170 10.1371/journal.pbio.0030170 15869325 PMC1088278

[B36] Chapman MA Leebens-Mack JH Burke JM Positive selection and expression divergence following gene duplication in the sunflower *CYCLOIDEA *gene family Mol Biol Evol 2008 25 7 1260 1273 10.1093/molbev/msn001 18390478

[B37] Wong WS Yang Z Goldman N Nielsen R Accuracy and power of statistical methods for detecting adaptive evolution in protein coding sequences and for identifying positively selected sites Genetics 2004 168 2 1041 1051 10.1534/genetics.104.031153 15514074 PMC1448811

[B38] Yang Z PAML: a program package for phylogenetic analysis by maximum likelihood Comput Appl Biosci 1997 13 5 555 556 9367129 10.1093/bioinformatics/13.5.555

[B39] Zhang JZ Nielsen R Yang ZH Evaluation of an improved branch-site likelihood method for detecting positive selection at the molecular level Mol Biol Evol 2005 22 12 2472 2479 10.1093/molbev/msi237 16107592

[B40] Yang Z Nielsen R Goldman N Pedersen AM Codon-substitution models for heterogeneous selection pressure at amino acid sites Genetics 2000 155 1 431 449 10790415 10.1093/genetics/155.1.431 PMC1461088

[B41] Sawyer S Statistical tests for detecting gene conversion Mol Biol Evol 1989 6 5 526 538 2677599 10.1093/oxfordjournals.molbev.a040567

[B42] Smith JM Analyzing the Mosaic Structure of Genes J Mol Evol 1992 34 2 126 129 1556748 10.1007/BF00182389

[B43] Bruen TC Philippe H Bryant D A simple and robust statistical test for detecting the presence of recombination Genetics 2006 172 4 2665 2681 10.1534/genetics.105.048975 16489234 PMC1456386

[B44] Jakobsen IB Easteal S A program for calculating and displaying compatibility matrices as an aid in determining reticulate evolution in molecular sequences Comput Appl Biosci 1996 12 4 291 295 8902355 10.1093/bioinformatics/12.4.291

[B45] Posada D Crandall KA Holmes EC Recombination in evolutionary genomics Annu Rev Genet 2002 36 75 97 10.1146/annurev.genet.36.040202.111115 12429687

[B46] Posada D Evaluation of methods for detecting recombination from DNA sequences: empirical data Mol Biol Evol 2002 19 5 708 717 11961104 10.1093/oxfordjournals.molbev.a004129

[B47] Posada D Crandall KA Evaluation of methods for detecting recombination from DNA sequences: computer simulations Proc Natl Acad Sci USA 2001 98 24 13757 13762 10.1073/pnas.241370698 11717435 PMC61114

[B48] Rice P Longden I Bleasby A EMBOSS: the European Molecular Biology Open Software Suite Trends Genet 2000 16 6 276 277 10.1016/S0168-9525(00)02024-2 10827456

[B49] Benjamini Y Hochberg Y Controlling the False Discovery Rate: a Practical and Powerful Approach to Multiple Testing J Royal Statis Soc B 1995 57 1 289 300

[B50] Storey JD Tibshirani R Statistical significance for genomewide studies Proc Natl Acad Sci USA 2003 100 16 9440 9445 10.1073/pnas.1530509100 12883005 PMC170937

[B51] Center for Disease Control and Prevention (CDC) *Salmonella *surveillance: Annual Summary, 2004 Atlanta, Georgia: US Department of Health and Human Services, CDC http://www.cdc.gov/ncidod/dbmd/phlisdata/salmonella.htm

[B52] Anisimova M Nielsen R Yang Z Effect of recombination on the accuracy of the likelihood method for detecting positive selection at amino acid sites Genetics 2003 164 3 1229 1236 12871927 10.1093/genetics/164.3.1229 PMC1462615

[B53] McClelland M Sanderson KE Spieth J Clifton SW Latreille P Courtney L Porwollik S Ali J Dante M Du FY Hou SF Layman D Leonard S Nguyen C Scott K Holmes A Grewal N Mulvaney E Ryan E Sun H Florea L Miller W Stoneking T Nhan M Waterston R Wilson RK Complete genome sequence of *Salmonella enterica *serovar typhimurium LT2 Nature 2001 413 6858 852 856 10.1038/35101614 11677609

[B54] Parkhill J Dougan G James KD Thomson NR Pickard D Wain J Churcher C Mungall KL Bentley SD Holden MTG Sebaihia M Baker S Basham D Brooks K Chillingworth T Connerton P Cronin A Davis P Davies RM Dowd L White N Farrar J Feltwell T Hamlin N Haque A Hien TT Holroyd S Jagels K Krogh A Larsen TS Leather S Moule S O'Gaora P Parry C Quail M Rutherford K Simmonds M Skelton J Stevens K Whitehead S Barrell BG Complete genome sequence of a multiple drug resistant *Salmonella enterica *serovar Typhi CT18 Nature 2001 413 6858 848 852 10.1038/35101607 11677608

[B55] Koonin EV Wolf YI Genomics of bacteria and archaea: the emerging dynamic view of the prokaryotic world Nucleic Acids Res 2008 36 6688 6719 10.1093/nar/gkn668 18948295 PMC2588523

[B56] Koski LB Morton RA Golding GB Codon bias and base composition are poor indicators of horizontally transferred genes Mol Biol Evol 2001 18 3 404 412 11230541 10.1093/oxfordjournals.molbev.a003816

[B57] Didelot X Achtman M Parkhill J Thomson NR Falush D A bimodal pattern of relatedness between the *Salmonella *Paratyphi A and Typhi genomes: convergence or divergence by homologous recombination? Genome Res 2007 17 1 61 68 10.1101/gr.5512906 17090663 PMC1716267

[B58] Hacker J Kaper JB Pathogenicity islands and the evolution of microbes Annual Rev Microbiol 2000 54 641 679 10.1146/annurev.micro.54.1.641 11018140

[B59] van Asten AJ van Dijk JE Distribution of "classic" virulence factors among *Salmonella *spp FEMS Immunol Med Microbiol 2005 44 3 251 259 10.1016/j.femsim.2005.02.002 15907446

[B60] Morgan E Campbell JD Rowe SC Bispham J Stevens MP Bowen AJ Barrow PA Maskell DJ Wallis TS Identification of host-specific colonization factors of *Salmonella enterica *serovar Typhimurium Mol Microbiol 2004 54 4 994 1010 10.1111/j.1365-2958.2004.04323.x 15522082

[B61] Kiss T Morgan E Nagy G Contribution of SPI-4 genes to the virulence of *Salmonella enterica* FEMS Microbiol Lett 2007 275 1 153 159 10.1111/j.1574-6968.2007.00871.x 17711458

[B62] Wood MW Jones MA Watson PR Hedges S Wallis TS Galyov EE Identification of a pathogenicity island required for *Salmonella *enteropathogenicity Mol Microbiol 1998 29 3 883 891 10.1046/j.1365-2958.1998.00984.x 9723926

[B63] Knodler LA Celli J Hardt WD Vallance BA Yip C Finlay BB *Salmonella *effectors within a single pathogenicity island are differentially expressed and translocated by separate type III secretion systems Mol Microbiol 2002 43 5 1089 1103 10.1046/j.1365-2958.2002.02820.x 11918798

[B64] Morgan E Rhen M, Maskell D, Mastroeni P, Threlfall J *Salmonella *Pathogenicity Islands Salmonella: Molecular Biology and Pathogenesis 2007 Norfolk: Horizon Bioscience 67 88

[B65] Folkesson A Lofdahl S Normark S The *Salmonella enterica *subspecies I specific centisome 7 genomic island encodes novel protein families present in bacteria living in close contact with eukaryotic cells Res Microbiol 2002 153 8 537 545 10.1016/S0923-2508(02)01348-7 12437215

[B66] Eswarappa SM Janice J Nagarajan AG Balasundaram SV Karnam G Dixit NM Chakravortty D Differentially evolved genes of *Salmonella *pathogenicity islands: insights into the mechanism of host specificity in *Salmonella* PLoS ONE 2008 3 12 e3829 10.1371/journal.pone.0003829 19050757 PMC2585142

[B67] Tracz DM Tabor H Jerome M Ng LK Gilmour MW Genetic determinants and polymorphisms specific for human-adapted serovars of *Salmonella enterica *that cause enteric fever J Clin Microbiol 2006 44 6 2007 2018 10.1128/JCM.02630-05 16757591 PMC1489402

[B68] Roca I Torrents E Sahlin M Gibert I Sjoberg BM NrdI essentiality for class Ib ribonucleotide reduction in *Streptococcus pyogenes* J Bacteriol 2008 190 14 4849 4858 10.1128/JB.00185-08 18502861 PMC2447006

[B69] Samant S Lee H Ghassemi M Chen J Cook JL Mankin AS Neyfakh AA Nucleotide biosynthesis is critical for growth of bacteria in human blood PLoS Pathog 2008 4 2 e37 10.1371/journal.ppat.0040037 18282099 PMC2242838

[B70] Buchmeier NA Libby SJ Xu Y Loewen PC Switala J Guiney DG Fang FC DNA repair is more important than catalase for *Salmonella *virulence in mice J Clin Invest 1995 95 3 1047 1053 10.1172/JCI117750 7883952 PMC441439

[B71] Negm RS Pistole TG The porin OmpC of *Salmonella *typhimurium mediates adherence to macrophages Can J Microbiol 1999 45 8 658 669 10.1139/cjm-45-8-658 10528398

[B72] Ho TD Slauch JM OmpC is the receptor for Gifsy-1 and Gifsy-2 bacteriophages of *Salmonella* J Bacteriol 2001 183 4 1495 1498 10.1128/JB.183.4.1495-1498.2001 11157969 PMC95030

[B73] Jiggins FM Adaptive evolution and recombination of *Rickettsia *antigens J Mol Evol 2006 62 1 99 110 10.1007/s00239-005-0080-9 16408241 PMC1800823

[B74] Pages JM James CE Winterhalter M The porin and the permeating antibiotic: a selective diffusion barrier in Gram-negative bacteria Nat Rev Microbiol 2008 6 12 893 903 10.1038/nrmicro1994 18997824

[B75] Alcaine SD Warnick LD Wiedmann M Antimicrobial resistance in nontyphoidal *Salmonella* J Food Protect 2007 70 3 780 790 17388077 10.4315/0362-028x-70.3.780

[B76] Medeiros AA O'Brien TF Rosenberg EY Nikaido H Loss of OmpC porin in a strain of *Salmonella *typhimurium causes increased resistance to cephalosporins during therapy J Infect Dis 1987 156 5 751 757 2821125 10.1093/infdis/156.5.751

