# Supplementary material for: Genome wide evolutionary analyses reveal serotype specific patterns of positive selection in selected Salmonella serotypes
Source: BMC Evol Biol. 2009 Nov 14;9:264. doi: 10.1186/1471-2148-9-264 (PMC2784778; doi:10.1186/1471-2148-9-264)
Supplement: Additional file 2 — Salmonella isolates (n = 42) used to verify genome wide positive selection and recombination patterns in four selected genes. [file 1471-2148-9-264-S2.DOC]

**Additional file 2.** *Salmonella* isolates (n=42) used to verify genome wide positive selection and recombination patterns in four selected genes

| **Isolate no. (FSL)^a^** | **Serotype** | **Source** | ***folk* PCR ampli-fication** | **STM3258 PCR ampli-fication** | ***sseC* PCR ampli-fication** | ***purE* PCR ampli-fication** |
| --- | --- | --- | --- | --- | --- | --- |
| S5-924 | 4, 5, 12:i:- | Cattle | Pos. | Neg. | Pos. | Pos. |
| S5-615 | 4, 5, 12:i:- | Cattle | Pos. | Neg. | Pos. | Pos. |
| S5-872 | Agona | Cattle | Pos. | Pos. | Neg. | Pos. |
| S5-366 | Dublin | Cattle | Pos. | Pos. | Pos. | Pos. |
| S5-549 | Havana | Cattle | Pos. | Pos. | Neg. | Pos. |
| S5-734 | Infantis | Cattle | Pos. | Pos. | Pos. | Pos. |
| S5-839 | Infantis | Cattle | Pos. | Pos. | Pos. | Pos. |
| S5-889 | Kentucky | Cattle | Pos. | Pos. | Neg. | Pos. |
| S5-947 | Mbandaka | Cattle | Pos. | Pos. | Neg. | Pos. |
| S5-559 | Montevideo | Cattle | Neg. | Pos. | Pos. | Pos. |
| S5-917 | Muenster | Cattle | Neg. | Pos. | Pos. | Pos. |
| S5-610 | Newport | Cattle | Pos. | Pos. | Pos. | Pos. |
| S5-611 | Newport | Cattle | Pos. | Pos. | Pos. | Pos. |
| S5-628 | Newport | Cattle | Pos. | Pos. | Pos. | Pos. |
| S5-715 | Newport | Cattle | Pos. | Pos. | Pos. | Pos. |
| S5-786 | T. Copenhagen | Cattle | Pos. | Pos. | Pos. | Pos. |
| S5-761 | Thompson | Cattle | Pos. | Pos. | Pos. | Pos. |
| S5-564 | Typhimurium | Cattle | Pos. | Pos. | Pos. | Pos. |
| S5-429 | Typhimurium | Cattle | Pos. | Pos. | Pos. | Pos. |
| S5-936 | Typhimurium | Cattle | Pos. | Neg. | Pos. | Pos. |
| S5-390 | 4, 5, 12:i:- | Human | Pos. | Neg. | Pos. | Pos. |
| S5-667 | Agona | Human | Pos. | Pos. | Neg. | Pos. |
| S5-529 | Anatum | Human | Pos. | Pos. | Pos. | Pos. |
| S5-373 | Braenderup | Human | Pos. | Pos. | Pos. | Pos. |
| S5-371 | Enteritidis | Human | Pos. | Pos. | Pos. | Pos. |
| S5-508 | Enteritidis | Human | Pos. | Pos. | Pos. | Pos. |
| S5-455 | Heidelberg | Human | Pos. | Pos. | Pos. | Pos. |
| S5-506 | Infantis | Human | Pos. | Pos. | Pos. | Pos. |
| S5-652 | Javiana | Human | Neg. | Pos. | Pos. | Pos. |
| S5-521 | Mbandaka | Human | Pos. | Pos. | Neg. | Pos. |
| S5-474 | Montevideo | Human | Neg. | Pos. | Pos. | Pos. |
| S5-504 | Muenchen | Human | Pos. | Pos. | Pos. | Pos. |
| S5-515 | Newport | Human | Pos. | Pos. | Pos. | Pos. |
| S5-524 | Newport | Human | Pos. | Pos. | Pos. | Pos. |
| S5-642 | Oranienburg | Human | Neg. | Pos. | Pos. | Pos. |
| S5-369 | Saintpaul | Human | Pos. | Pos. | Pos. | Pos. |
| S5-412 | Thompson | Human | Pos. | Pos. | Pos. | Pos. |
| R6-540 | Typhi | Human | Pos. | Pos. | Pos. | Pos. |
| R6-623 | Typhi | Human | Pos. | Pos. | Pos. | Pos. |
| S5-370 | Typhimurium | Human | Pos. | Pos. | Pos. | Pos. |
| S5-531 | Typhimurium | Human | Pos. | Pos. | Pos. | Pos. |
| S5-659 | Urbana | Human | Neg. | Pos. | Pos. | Pos. |

^a^Full isolate designations carry the “FSL” prefix (e.g., FSL S5-924); additional information on all isolates is available at [www.pathogentracker.net](http://www.pathogentracker.net/), which can be searched with the full isolate designation (e.g., FSL S5-924)
