# Supplementary material for: Genome wide evolutionary analyses reveal serotype specific patterns of positive selection in selected Salmonella serotypes
Source: BMC Evol Biol. 2009 Nov 14;9:264. doi: 10.1186/1471-2148-9-264 (PMC2784778; doi:10.1186/1471-2148-9-264)
Supplement: Additional file 3 — PCR conditions and primers for the four genes that were used to verify genome wide positive selection and recombination patterns in an additional 42 Salmonella isolates. [file 1471-2148-9-264-S3.DOC]

**Additional file 3. PCR conditions and primers for the four genes that were used to verify genome wide positive selection and recombination patterns in an additional 42 *Salmonella*** isolates

| Gene | Forward Primer (5’ to 3’) | Reverse Primer (5’ to 3’) | Denaturation temperature (time) | Annealing temperature (time) | Extension temperature (time) | Cycles |
| --- | --- | --- | --- | --- | --- | --- |
| *folk-2* | TTGGCGCTAAAGCAGTAAAC | CCGCGAGGGCACCGTATG | 95^0^C (45 sec) | 52^0^C (45 sec) | 72^0^C (45 sec) | 30 |
| STM3258 | TTCAGGATGTTCTGCGCGG | ACCGCACCGCCACAGGCC | 95^0^C (45 sec) | 55^0^C (45 sec) | 72^0^C (45 sec) | 30 |
| *sseC*-1^a^ | CAGTGAACATCAAATTCTCCG | CCATATAAGCTACGCCGC | 95^0^C (1 min) | 52^0^C (45 sec) | 72^0^C (45 sec) | 30 |
| *sseC*-2^a^ | ATAAAGCGCGTAAAGCGGG | CGCTTCTTTTGCCATGGC | 95^0^C (1 min) | 52^0^C (45 sec) | 72^0^C (45 sec) | 30 |
| *sseC*-3^a^ | AACGTGAAATGGCTATGGCG | TGTTAACAAGGAAGGCGC | 95^0^C (1 min) | 52^0^C (45 sec) | 72^0^C (45 sec) | 30 |
| *purE* | ATCGCCTTCCCCCTGTAAATGTTC | TAATGACGCTCTGCTGTACCGGC | 95^0^C (45 sec) | 55^0^C (45 sec) | 72^0^C (45 sec) | 30 |

^a^*sseC* was amplified with three separate primer sets designated *sseC*-1, *sseC*-2, and *sseC*-3
